# Estimation of a Minimum Allowable Structural Strength Based on Uncertainty in Material Test Data

**DOI:** 10.6028/jres.126.036

**Published:** 2021-12-07

**Authors:** Jeffrey T. Fong, N. Alan Heckert, James J. Filliben, Pedro V. Marcal, Stephen W. Freiman

**Affiliations:** 1 National Institute of Standards and Technology, Gaithersburg, MD 20899, USA; 2 MPACT Corp., Oak Park, CA 91377, USA; 3 Freiman Consulting, Potomac, MD 20854, USA

**Keywords:** aluminum oxide, Anderson-Darling criterion, ASTM C1239-07, borosilicate crown BK-7 glass, chi-square criterion, DATAPLOT, failure strength test, goodness-of-fit, high-strength steels, Kolmogorov-Smirnov criterion, lognormal, maximum likelihood method, model selection, normal, probability plot correlation coefficient, probability plot correlation coefficient criterion, silicon nitride, statistical data analysis, structural reliability, uncertainty quantification, Weibull distribution

## Abstract

Three types of uncertainties exist in the estimation of the minimum fracture strength of a full-scale component or structure size. The first, to be called the “model selection uncertainty,” is in selecting a statistical distribution that best fits the laboratory test data. The second, to be called the “laboratory-scale strength uncertainty,” is in estimating model parameters of a specific distribution from which the minimum failure strength of a material at a certain confidence level is estimated using the laboratory test data. To extrapolate the laboratory-scale strength prediction to that of a full-scale component, a third uncertainty exists that can be called the “full-scale strength uncertainty.” In this paper, we develop a three-step approach to estimating the minimum strength of a full-scale component using two metrics: One metric is based on six goodness-of-fit and parameter-estimation-method criteria, and the second metric is based on the uncertainty quantification of the so-called A-basis design allowable (99 % coverage at 95 % level of confidence) of the full-scale component. The three steps of our approach are: (1) Find the “best” model for the sample data from a list of five candidates, namely, normal, two-parameter Weibull, three-parameter Weibull, two-parameter lognormal, and three-parameter lognormal. (2) For each model, estimate (2a) the parameters of that model with uncertainty using the sample data, and (2b) the minimum strength at the laboratory scale at 95 % level of confidence. (3) Introduce the concept of “coverage” and estimate the fullscale allowable minimum strength of the component at 95 % level of confidence for two types of coverages commonly used in the aerospace industry, namely, 99 % (A-basis for critical parts) and 90 % (B-basis for less critical parts). This uncertainty-based approach is novel in all three steps: In step-1 we use a composite goodness-of-fit metric to rank and select the “best” distribution, in step-2 we introduce uncertainty quantification in estimating the parameters of each distribution, and in step-3 we introduce the concept of an uncertainty metric based on the estimates of the upper and lower tolerance limits of the so-called A-basis design allowable minimum strength. To illustrate the applicability of this uncertainty-based approach to a diverse group of data, we present results of our analysis for six sets of laboratory failure strength data from four engineering materials. A discussion of the significance and limitations of this approach and some concluding remarks are included.

## 1 Introduction

**Fig. 1:**
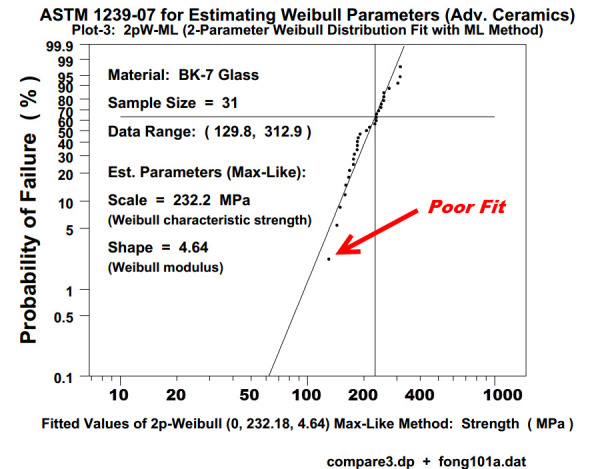
A two-parameter Weibull plot of a set of 31 biaxial test data for the ultimate tensile strength of a BK-7 glass [6].

One of the most difficult questions in structural engineering design and failure analysis is how to best fit a set of fracture, yield, or ultimate strength test data. In the standard practice for advanced ceramic materials recommended by ASTM International [1], the two-parameter (2p) Weibull distribution with a zero-location parameter was used (see, *e.g*., Fig. 1). The choice of the 2p Weibull, rather than other models such as the three-parameter (3p) Weibull, or 2p or 3p lognormal, *etc*., may conceivably be attributed to a lack of (a) efficient computational codes for parameter estimation for alternative distributions in the literature, and (b) easy-to-use criteria for choosing the "best" distribution among competing and equally reasonable one. 

While recognizing that the zero-location feature of a 2p Weibull model might be acceptable for modeling the life of a product [2, 3, 4], one cannot help but observe that a 2p Weibull is physically unrealistic for modeling the minimum strength of an engineering material, because it assumes that among all possible samples of an engineering material, one will likely fail a simple tensile strength test near the zero load. It is also unduly conservative when recommended as the so-called A-basis (99% coverage) for critical and B-basis (90% coverage) for less-critical structural design allowable in aerospace industry [5].

**Fig. 2:**
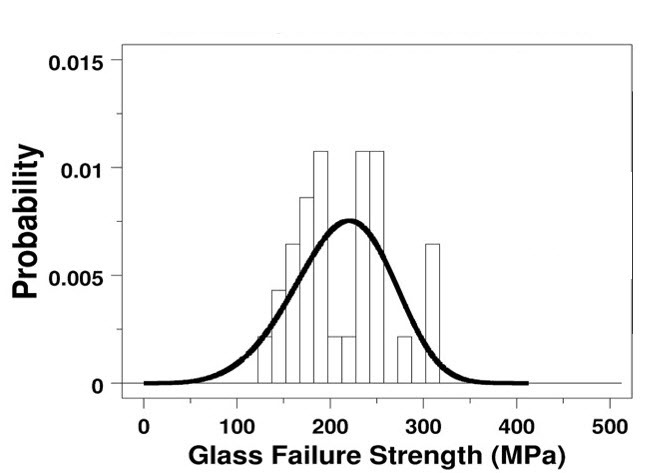
Histogram and 2p Weibull probability density function of a set of 31 biaxial test data for the ultimate tensile strength of a BK-7 glass [6] Using the maximum likelihood method of parameter estimation, we found the scale parameter is 232.2 MPa, and the shape is 4.64 (for *n* = 31).

**Fig. 3:**
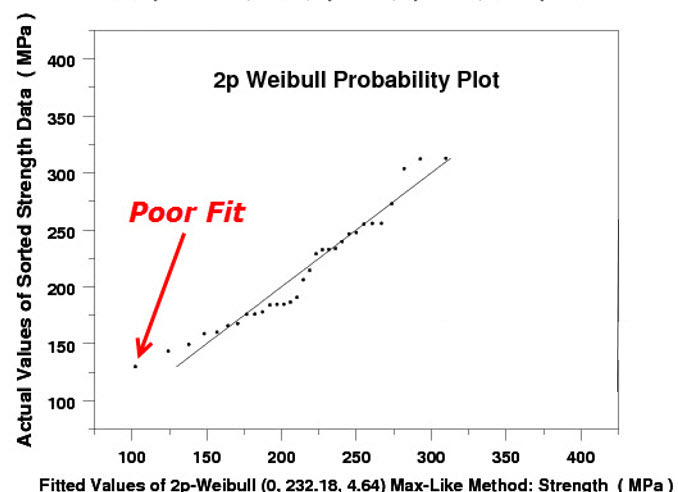
BK-7 glass: A 2p Weibull probability plot of the glass strength data *vs*. predicted values.

**Fig. 4:**
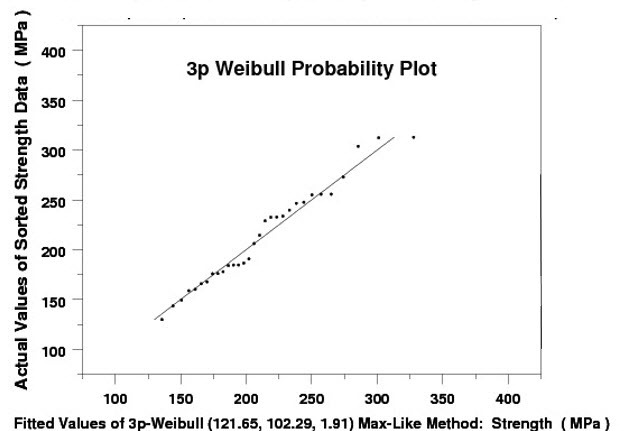
BK-7 glass: A 3p Weibull probability plot of the glass strength data *vs*. predicted values.

**Fig. 5:**
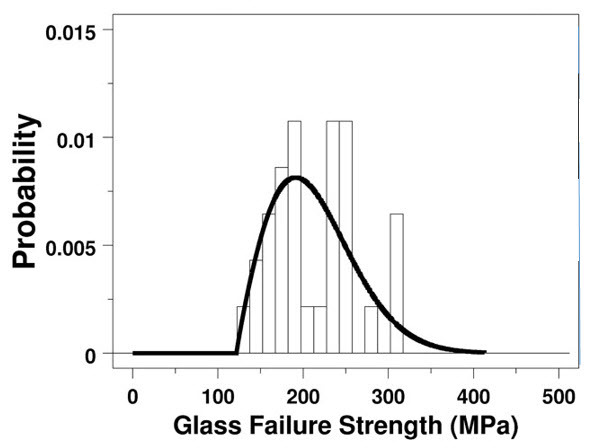
BK-7 glass: Histogram and 3p Weibull probability density function for the same strength data. Using the ML method of parameter estimation, we found the location parameter is 121.7 MPa, the scale is 102.3 MPa, and the shape is 1.91 (for *n* = 31).

To illustrate the need for a re-examination of the basis for choosing the “best” model of a set of tensile strength data, we applied the ASTM recommended practice C1239-07 [1] to a set of 31 ring-on-ring test data for an aircraft window material, borosilicate crown BK-7 glass (see Appendix A, Data Set No. 1, which is based on Fuller *et al*. [6]). In Figs. 1 and 2, we present the 2p Weibull probability plot and the histogram and probability density function, respectively, of the 31 data point set according to ASTM 1239-07. In this exercise, we used the maximum likelihood (ML) method [7, 8] in a statistical analysis code (written in DATAPLOT [9]) to estimate the parameters of a 2p Weibull distribution. In Fig. 3, we used the same code to make a quantile-quantile (QQ) plot of the same set of data *vs*. the predicted values based on a 2p Weibull. In both Figs. 1 and 3, we observe that near the lower values of the 31 data point set, the fit is not so good. On the other hand, when we used the same code to estimate the parameters of a 3p Weibull, the fit is remarkably good, as shown in Figs. 4 and 5.

To improve the 2p Weibull methodology recommended in the ASTM standard practice C1239-07 [1] for reporting strength data of ceramic materials, we developed a set of new tools not only for a set of five distributions (normal, 2p Weibull, 3p Weibull, 2p lognormal, 3p lognormal), but also for a broader class of materials that includes ceramics, metal alloys and composites. Our approach consists of three steps (see Sec. 2, 3, and 4, respectively):

Step 1. Model Selection. Find the “best” model for the sample data from a list of five candidates, namely, normal, 2p Weibull, 3p Weibull, 2p lognormal, and 3p lognormal. Analysis results for glass data are given in Sec. 2, Table 1. 

Step 2. Laboratory-Scale Statistical Analysis. Estimate with uncertainty quantification for each model the parameters of that model using the sample data and the lower and upper bounds of the minimum strength at the laboratory scale for a 95% level of confidence. Analysis results for glass data are given in Sec. 3, Table 2.

Step 3. Full-Scale Statistical Analysis. We introduce in Sec. 4 the concept of “coverage” and the classical theory of tolerance limits to estimate the minimum allowable strength, also with uncertainty quantification, of a full-scale structure at 95% confidence level and two specific sizes of coverage, namely 99% (also known as the A-basis) and 90% (known as the B-basis).

In Sec. 5, we show the results of applying this new approach to six data sets (see Appendix A) from four engineering materials. The significance of our approach and some concluding remarks are given in Sec. 6 and Sec. 7, respectively. In addition to Sec. 3, Table 2 (results for Data Set No. 1), we attach in Appendices C through the complete numerical results of the application of our approach for Data Set Nos. 2 through 6, respectively.

## 2 Model Selection (Step 1 of 3)

We began the development of our new approach by considering five candidate models and selecting the "best" over two parameter-estimation (PE) methods and four goodness-of-fit (GoF) criteria. The five candidate models were:

(1) Model 1: normal (N),
(2) Model 2: two-parameter Weibull (2*p*W),
(3) Model 3: three-para. Weibull (3*p*W).
(4) Model 4: two-para. lognormal (2*p*LN),
(5) Model 5: three-para. lognormal (3*p*LN), and

The two-parameter estimation (PE) methods were:

(1) PE Method No. 1: Maximum likelihood (ML) method (see, *e.g*., Bury [10, pp. 161-168], Aldrich [7], and Anderson [8]).
(2) PE Method No. 2: Probability plot correlation coefficients (PPCC) method (see, Filliben [11, 12], Looney and Gulledge Jr. [13], and Vogel [14]).

The four GoF criteria are:

(1) GoF Criterion 1. Anderson Darling (AD) test (see Anderson and Darling [15, 16]).
(2) GoF Criterion 2. Kolmogorov-Smirnov (KS) test (see, *e.g*., Bury [10, pp. 204-208]).
(3) GoF Criterion 3. Chi square (CS) test (see, *e.g*., Bury [10, pp. 196-203]).
(4) GoF Criterion 4. Probability plot correlation coefficient (PC) test (see, *e.g*., Filliben [11, 12], Looney and Gulledge Jr. [13], and Vogel [14]).

To compute the GoF statistics for each model, we adopted the following six GoF-PE scenarios:

(1) GoF-PE Scenario 1 (CS-1): GoF Criterion 3 (CS) with PE Method No. 1 (ML).
(2) GoF-PE Scenario 2 (AD-1): GoF Criterion 1 (AD) with PE Method No. 1 (ML).
(3) GoF-PE Scenario 3 (KS-1): GoF Criterion 2 (KS) with PE Method No. 1 (ML).
(4) GoF-PE Scenario 4 (AD-2): GoF Criterion 1 (AD) with PE Method No. 2 (PC).
(5) GoF-PE Scenario 5 (KS-2): GoF Criterion 2 (KS) with PE Method No. 2 (PC).
(6) GoF-PE Scenario 6 (PC-2): GoF Criterion 4 (PC) with PE Method No. 2 (PC).

To test our methodology, we obtained from the literature a total of six data sets (see Appendix A) for four materials, namely, BK-7 glass (Data Set No. 1), silicon nitrate (Data Set No. 2 and Data Set No. 3 for two different test methods), aluminum oxide (Data Set No. 4), and a high-strength steel (Data Set- No. 5 and Data Set No. 6 for two different temperature environments). Based on formulas in the statistics literature [17–20], we wrote an analysis code in DATAPLOT to capture the GoF statistics for the six GoF-PE scenarios of all five models for each of the six ultimate strength data sets as listed in Appendix A. Results of the analysis for all five models with their raw GoF statistics for each of the six data sets are given in Appendix B. 

An examination of the raw GoF statistics for each data set in Appendix B showed a qualitative difference between those of the first five GoF-PE scenarios (CS-1, AD-1, KS-1, AD-2 and KS-2) and the sixth scenario (PC-2): namely, the former interprets a smaller statistic to be a better fit, whereas the latter demands that a larger statistic is better. This requires us to develop two sets of normalization formulas as follows:

(1) For each of the five scenarios, CS-1, AD-1, KS-1, AD-2, and KS-2, let *x_i_*, (*i* = 1, …, 5), be the GoF statistics of the five candidate models being considered for selection, and let *x_max_* and *x_min_* be the maximum and minimum of the five statistics, *x_i_*, (*i* = 1, …, 5), respectively. The normalized statistic of *x_i_* , (*i* = 1, …, 5), to be denoted by *Nx_i_*, (*i* = 1, …, 5), is defined as follows:

*Nx_i_* = (*x_i_* - *x_max_*) / (*x_min_* - *x_max_*), (*i* = 1, …, 5). (1)

(2) For the sixth scenario, PC-2, let *y_i_*, (*i* = 1, …, 5), be the GoF statistics of the five candidate models being considered for selection, and let *y_max_* and *y_min_* be the maximum and minimum of the five statistics, *y_i_*, (*i* = 1, …, 5), respectively. The normalized statistic of *y_i_*, (*i* = 1, …, 5), to be denoted by *Ny_i_* , (*i* = 1, …, 5), is defined as follows:

*Ny_i_* = (*y_i_* - *y_min_*) / (*y_max_* - *y_min_*), (*i* = 1, …, 5). (2)

Using the average of the normalized GoF statistics as a metric for ranking (with 1 being good, 0 being poor) as shown in the second row from the bottom of Table 1, we observe that the 3pW model (metric 1 = 1.00) ranks first among all five candidate models for Data Set No. 1 (BK-7 glass).

**Table 1:** Data Set No. 1: BK-7 glass–Goodness-of-fit statistics for five candidate models.

No.	GoF- PE Combo	Goodness-of-Fit (GoF) and Parameter Estimation (PE) Method Statistical Analysis Scenario Description	Model 1 Normal	Model 2 Two- parameter Weibull	Model 3 Three- parameter Weibull	Model 4 Two- parameter lognormal	Model 5 Three- parameter lognormal
1	CS-1	Chi square (CS) criterion + ML method (PE-1) gives GoF statistics = (Note: Small is good.) Normalized CS-1 statistics between 0 and 1 (best) =	13.72 0.00	13.70 0.01	10.61 1.00	11.65 0.67	11.91 0.58
2	AD-1	Anderson-Darling (AD) + ML method (PE-1) gives GoF statistics = (Note: Small is good.) Normalized AD-1 statistics between 0 and 1 (best) =	0.532 0.25	0.597 0.00	0.338 1.00	0.389 0.80	0.398 0.77
3	KS-1	Kolmogorov-Smirnov (KS) + ML method (PE-1) gives GoF statistics = (Note: Small is good.) Normalized KS-1 statistics between 0 and 1 (best) =	0.151 0.06	0.153 0.00	0.117 1.00	0.122 0.86	0.129 0.67
4	AD-2	Anderson-Darling (AD) + probability plot correlation coefficient (PC) method (PE-2) gives GoF statistics = (Note: Small is good.) Normalized AD-2 statistics between 0 and 1 (best) =	0.513 0.00		0.318 1.00		0.374 0.71
5	KS-2	Kolmogorov-Smirnov (KS) + probability plot correlation coefficient (PC) method (PE-2) gives GoF statistics = (Note: Small is good.) Normalized KS-2 statistics between 0 and 1 (best) =	0.149 0.00		0.114 1.00		0.124 0.71
6	PC-2	Probability plot correlation coefficient (PC) criterion + PC method (PE-2) gives GoF statistics = (Note: Large is good.) Normalized PC-2 statistics between 0 and 1 (best) =	0.980 0.00		0.988 1.00		0.986 0.75
		Column sum of all normalized statistics =	0.31	0.01	6.00	2.33	4.19
		Average of normalized GoF statistic from 0 to 1 =	0.05	0.00 (worst)	1.00 (best)	0.78	0.60
		GoF ranking (1 being best, and 5 being worst)	4	5	1	2	3

## 3 Parameter Estimation and Minimum Strength at Laboratory Scale (Step 2 of 3)

In the last section (Step 1: Model Selection), we applied a multiple PE-method-GoF scenario technique and an elementary two-step normalization algorithm to develop a composite GoF index as a “metric” for ranking the five candidate models according to which one “best” fits a given set of strength data. Having chosen the 3pW as the “best- fit” model, we wrote a second analysis code that gave not only the point estimates of the location, scale, and shape parameters of the 3pW, but also their standard errors, upper and lower limits at various confidence intervals (which are useful in this step, step 2, to estimate the minimum strength at laboratory sample-size scale), the one-sided tolerance limits at 95% confidence level for 12 coverages varying from 90% to 99.99999999% (which are useful in the next step, step 3, that is designed to estimate the upper and lower limits of the minimum strength at full-scale component size). 

**Fig. 6:**
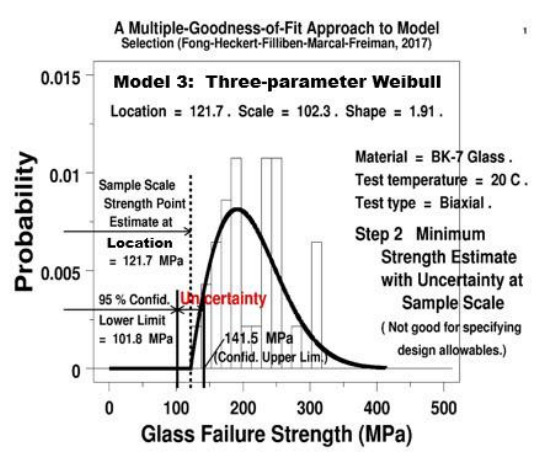
BK-7 glass: Histogram and fitted 3p Weibull probability density function for the same strength data with lower and upper 95% confidence limits for the location parameter at the sample scale.

In order to clarify the difference between the two estimates of the minimum strength, or the location parameter (for a 3pW model), one being at the laboratory sample-size scale, and the other being at the full-scale component size, we will address in this section only the second step (laboratory scale) of our methodology by using the first half of the analysis results (without coverages), that is, based on the second analysis code, “3pW_0.05x.dp.” Figure 6 shows a plot of the results for Data Set No. 1 using the 3pW model to estimate the minimum strength and its uncertainty at laboratory scale. The complete results of our three-step analysis for all five models for Data Set No. 1 are tabulated in Table 2.

**Table 2:** Estimates of minimum strength at laboratory-scale and full-scale sizes for five candidate models based on Data Set No. 1 (BK-7 glass) at 20 °C (biaxial strength test).

	Model 1 Normal	Model 2 2p Weibull	Model 3 3p Weibull	Model 4 2p lognormal	Model 5 3p lognormal
Laboratory-Scale					
Composite normalized GoF statistic (metric 1) (Ranges from 0 to 1, worst to best.)	0.05	0 (worst)	1.0 (best)	0.78	0.60
Parameter 1 (Location) Point estimate of location, Standard deviation of location	212.4 9.0	None. None.	121.7 12.1	None. None.	41.8 77.3
One-sided 95% confidence minimum strength at sample scale = (lower limit, point estimate, upper limit)	(95.6, 130.2, 153.0)	(96.1, 122.3, 148.6)	(101.8, 121.7, 141.5)	(119.5, 140.6, 156.5)	(-85.0, 41.8, 169.0)
Parameter 2 (Scale) Point estimate of scale, Standard deviation of scale	50.0 6.5	232.2 9.5	102.3 11.8	206.9 8.9	163.6 81.1
Parameter 3 (Shape) Point estimate of shape, Standard deviation of shape	None. None.	4.64 0.65	1.91 0.45	0.24 0.03	0.29 0.15
Full-Scale Size					
95% confidence, 99% coverage A-basis design allowable (AbDA) Uncertainty (metric 2) (Note: Small is good.)	38% (worst)	30%	11% (best)	16%	19%
95% confidence, 99% coverage minimum strength at full scale (lower tolerance limit or A-basis of design, mean estimate, upper tolerance limit)	(51.7, 96.1, 124.1)	(60.0, 86.1, 112.5)	(116.3, 130.9, 145.5)	(97.2, 119.8, 136.6)	(101.3, 124.8, 148.4)
95% confidence, 90% coverage minimum strength at full scale (lower tolerance limit or B-basis of design, mean estimate, upper tolerance limit)	(118.6, 148.4, 168.8)	(117.8, 142.9, 168.0)	(139.5, 153.2, 166.9)	(133.1, 153.1, 168.5)	(138.7, 154.4, 170.1)

## 4 Minimum Strength with Uncertainty at Full-Scale Component Size (Step 3 of 3)

In the last section (Step 2: Laboratory-Scale Minimum Strength Estimation), we introduced a DATAPLOT code to compute not only parameters with uncertainty quantification, but also one-sided tolerance limits for 12 coverages ranging from 90% to 99.99999999%. It turns out that the estimates of the tolerance limits are exactly what we need for step 3 of our new methodology. Here, we need to introduce a new concept, namely, “coverage.” As shown by Nelson, *et al.* [20, pp. 179–180], when the true mean, *μ*, and standard deviation, *σ*, of a normal distribution are not known, the so- called (1-*α*) 100% prediction interval is given by the following expression:

*ȳ ± t (α / 2 ; n - 1) s √ (1 + 1/n),* (3)

where *ȳ* is the estimated mean, *s* is the estimated standard deviation, *n* is the sample size, *t* is the well-known Student’s distribution function, and *α* is the quantity associated with the confidence level given by (1 - α) 100%. For example, a 95% confidence level is specified by *α* = 0.05. For engineers dealing with material testing data, the estimated prediction interval given in Eq. (3) for a normally distributed sample data set is valid only at the sample-scale size. 

To extrapolate the sample-scale size estimate to a larger scale, we need the concept of the so-called “coverage,” *p*, or, the proportion of the population that is covered by a new statistical interval known as the “tolerance interval,” (see again, *e.g*., Nelson, *et al.* [20, pp. 179–180]). The upper limit and lower limit of the tolerance interval are known as the upper tolerance limit (UTL) and lower tolerance limit (LTL), respectively. It is the one-sided LTL for a given coverage, *p*, and (1 - *α*) 100% confidence level that engineers are interested in for finding the design allowable of a minimum strength for a given structural material. The theory of tolerance intervals for a normal population is well-known in the literature (see, *e.g*., Prochan [21], Natrella [22], and Nelson, *et al*. [20]). As shown by Nelson, *et al*. [20], the tolerance interval for a normal population with a given estimated mean, *ȳ*, and standard deviation, *s*, is given below:

*ȳ ± r u s,* (4)

where the factor, *r* (*n, p*), depends on the sample size, *n*, and the coverage, *p*, and the factor, *u (df, γ)*, depends on the degrees of freedom, *df*, defined by *n -* 1, and the confidence level, *γ*, defined by 1 *- α* . Both factors for limited ranges of *n, p*, and *γ*, are given in tables of Natrella [22] for two-sided and one-sided LTL, and tables of Nelson, *et al*. [20] for two-sided LTL for a normal population only.

**Fig. 7:**
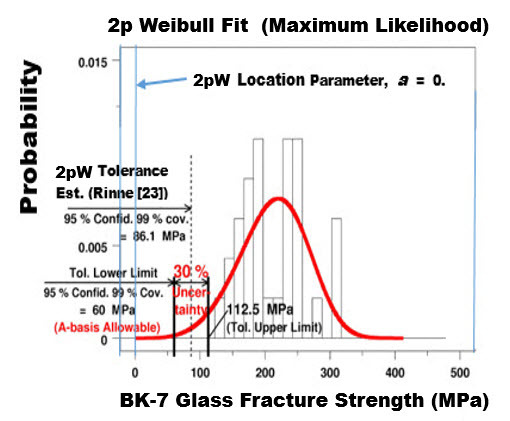
BK-7 glass: Histogram and fitted 2p Weibull probability density function for the same strength data with lower and upper tolerance limits at 95% confidence and 99% coverage (A-basis).

**Fig. 8:**
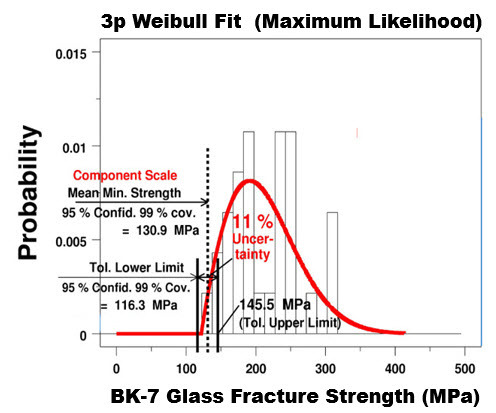
BK-7 glass: Histogram and fitted 3p Weibull probability density function for the same strength data with lower and upper tolerance limits at 95% confidence and 99% coverage (A-basis).

When the underlying distribution is a 3pW, the tolerance interval for a 3pW population can be estimated using formulas given by Rinne [23, pp. 585–600] and implemented in a computer code we wrote in DATAPLOT [9]. Plots of the tolerance limits at 99% coverage using the 2pW and 3pW models are given in Figs. 7 and 8, respectively. If we define a new metric, “uncertainty,” as the ratio of the quantity (upper limit– lower limit) to two times the mean minimum strength, then the uncertainties of the 2pW and 3pW minimum strength are given as 30% and 11%, respectively.

For completeness, we list below the formulas we used (based on Rinne [23]) for implementing the necessary computation in our DATAPLOT code. Following Rinne [23], we used the ML method to estimate the three Weibull parameters, namely, *a* (location), *b* (scale), and *c* (shape). Let *P* be percentile, and let the coverage, *p*, be given by 1*- P*. For example, for *p* = 0.99 or a 99% coverage, *P* = 0.01. Instead of Eq. (4) that is applicable only for a normal distribution model, the one-sided lower tolerance interval for a 3pW population has the following two-term form:

$\hat{x}P,L=\;{\hat{x}}_{P}-u_{1-\alpha /2}\;\surd (A\;Var\left(\;{\hat{x}}_{P}\;\right)\;)$, (5)

where the first term is given by

${\hat{x}}_{P}=a+b\;{\left[\;-\ln\left(\;1-P\;\right)\right]}^{1/c}$, (6)

and the second term is the product of two factors, the first of which is available from a normal distribution table, and the second of which is given by

$A\;Var\left({\hat{x}}_{P}\right)=\frac{b^{2}}{nD}\;\{\;\frac{B}{{\left(c-1\right)}^{2}}-\frac{2\beta^{1/c}}{c\left(c-1\right)}\left(H+F\;ln\beta \right)+\frac{\beta^{1/c}}{c^{2}}\left[A-2G\;ln\beta +{\left(ln\beta \right)}^{2}\right]\;\}$, (7)

where *β* =- ln(1- *P*), and *A, B, D, F, G,* and *H* are defined in Eqs. (11.12b-h) of Rinne [23].

As a special case when we let the location parameter, *a*, be zero in Eq. (6), we obtain the one-sided lower tolerance interval for a 2pW population using the same set of Eqs. (5), (6), and (7), shown above, and Eqs. (11.12b-h) of Rinne [23].

In Table 2, we show the values of the so-called A-basis (99% coverage) design allowable (AbDA) uncertainty for all five models and observe that the AbDA metric correlates well with the composite GoF metric (metric 1). This completes our three-step approach as an alternative to the ASTM standard C1239-07 [1]. 

## 5 Application of the Three-Step Methodology to Six Sets of Minimum Strength Data

To show that our new approach is applicable not only to glass, as we did in the previous three sections, but also to other ceramic or metallic materials, we applied the three-step methodology to five more data sets (see Data Set Nos. 2 through 6 in Appendix A). Those data sets came from Duffy, *et al.* [24], Quinn [25], and NRIM [26], which provided data sets for two more ceramic materials and a metal alloy (a high-strength steel used in the World Trade Center Towers). The complete analysis results for all six sets of data including Data Set No. 1 (glass) are summarized in Table 3.

For completeness, we attach the raw GoF statistics for all six data sets in Appendix B, and the results of the three-step analysis for the remaining five data sets, Data Set Nos. 2 through 6, in Appendices C through G, respectively.

**Fig. 9:**
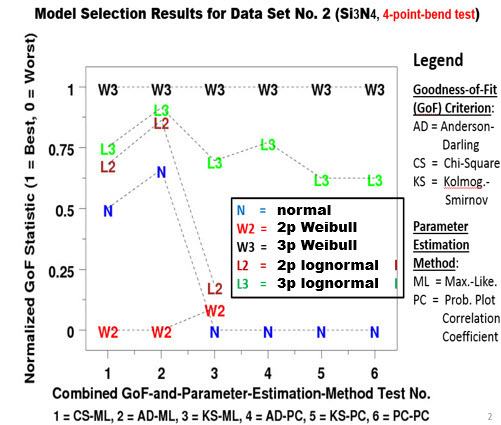
Silicon nitride **(**Si_3_N_4_) with a four-point bend test: Model selection results (Data Set No. 2).

**Fig. 10:**
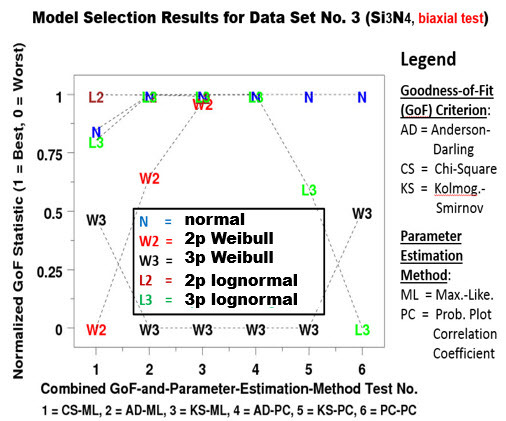
Silicon nitride **(**Si_3_N_4_) with a biaxial test: Model selection results (Data Set No. 3).

It is interesting to note that Data Set Nos. 2 and 3 are for the same material, silicon nitride (Si_3_N_4_), based on two different tests, namely the four-point bend test (Data Set No. 2), and the biaxial pressurized disk test (Data Set No. 3). Plots of the normalized GoF statistic *vs*. the six GoF test scenarios in Figs. 9 and 10 for the two data sets clearly shows that the selection of the best-fit model for silicon nitride depends on the test from which the data were generated. If it is from a four-point bend test, the choice is 3pW. If it is from a biaxial test, the choice is normal. The 2pW function is, nevertheless, not a good choice in either case. For brevity, similar plots for Data Set Nos. 1, 4, 5, and 6 are not included in this paper.

**Table 3:** Model selection results for six data sets of fracture strength of four materials.

Data Set No., Material, Temperature	Sample Size Mean Standard- Deviation	Metric No.	Model 1 Normal	Model 2 Two- parameter Weibull	Model 3 Three- parameter Weibull	Model 4 Two- parameter lognormal	Model 5 Three- parameter lognormal
1. BK-7 glass 20 °C	31 212.4 MPa 50.0 MPa	Metric 1 Metric 2 Rank based on metric 1	0.05 38% 4	0 30% 5 (worst)	1.0 11% 1 (best)	0.78 16% 2	0.60 19% 3
2. Silicon nitride (four-point bend) 20 °C	27 733.2 MPa 77.7 MPa	Metric 1 Metric 2 Rank based on metric 1	0.19 11% 4	0.03 15% 5 (worst)	1.0 4% 1 (best)	0.58 13% 3	0.73 7% 2
3. Silicon nitride (bi-axial test) 20 °C	32 688.7 MPa 63.1 MPa	Metric 1 Metric 2 Rank based on metric 1	0.97 8% 1 (best)	0.54 12% 4	0.16 12% 5 (worst)	1.0^a^ 7%^a^ 2^a^	0.73 10% 3
4. Al. oxide 20 °C	30 444.0 MPa 52.1 MPa	Metric 1 Metric 2 Rank based on metric 1	1.0 12% 1 (best)	0.88 16% 3	0.32 13% 4	0.97 9% 2	0.12 57% 5 (worst)
5. High- strength steel, 20 °C	21 638.3 43.3 MPa	Metric 1 Metric 2 Rank based on metric 1	0.28 7% 4	0 12% 5 (worst)	1.0 3% 1 (best)	0.69 6% 3	0.85 4% 2
6. High- strength steel, 600 °C	21 300.6 MPa 26.2 MPa	Metric 1 Metric 2 Rank^b^ based on metric 2	0.64 10% 4	0.28 15% 5 (worst)	0.58 4% 1 (best)	0.93^b^ 8% 3	0.49 6% 2

^a^ The assumption of a zero-location parameter precludes model 4 from being selected as rank 1.

^b^ In this special case when we disqualified model 4 for being selected as rank 1 based on metric 1 and found the metric 1 values of model 1 and model 3 too close to call, we switched to the use of metric 2 for ranking the model selection process.

## 6 Significance and Limitations of the Three-Step Minimum Strength Modeling Approach

The proposed three-step approach outlined in this paper is novel in each of its three steps. In step 1 (Model Selection), we developed a composite normalized GoF statistic named metric 1 to rank and select the “best” model. In step 2 (Laboratory-Scale Modeling), we introduced a method for quantifying the uncertainty of the parameters of each distribution by estimating both their mean value and standard deviation. In step 3 (Component or Structural Full-Scale Modeling), we formulated the concept of an uncertainty metric named metric 2 based on the estimates of the upper and lower tolerance limits of the so-called A-basis design allowable minimum strength. In Table 4, we show the significance of our new approach by comparing the A-basis design allowable minimum strength for the six data sets using either the 2pW (ASTM C1239-07 [1]), or the best-fit choice from our approach. A word of caution needs to be said about the limitations of our approach. First of all, we assumed that the test data are unimodal, and we chose our candidate distributions also to be unimodal, so it is not clear if our approach will be useful if the test data set is not unimodal. Second, we only chose a small set of models, namely five (normal, 2pW, 3pW, 2pLN, and 3pLN), to work with, and there may be many other distributions that could better fit the data. Nevertheless, since the ASTM C1239-07 suffers the same limitations as outlined above, our approach provides a new and more rational alternative to the current practice.

**Table 4:** Comparison of the A-basis design allowable minimum strength (MPa) selected from the 2pW model (ASTM) approach *vs.* our approach by making the best choice among five models according to a goodness-of-fit or tolerance limit uncertainty metric.

	"A-Basis" Design Allowable Minimum Strength (MPa) (95% confidence, 99% coverage)			
Data Set (DS) No., Material Name, Temperature (Type of Strength Test)	2p Weibull (ASTM) Approach	Our Approach	Difference		
DS-1. BK-7 glass at 20 °C (biaxial test)	60.0	116.3	+ 94%		
DS-2. Silicon nitride at 20 °C (four-point bend test)	414.2	590.5	+ 43%		
DS-3. Silicon nitride at 20 °C (biaxial test)	432.1	487.1	+ 13%		
DS-4. Aluminum oxide at 20 °C (uniaxial test)	239.2	275.7	+ 15%		
DS-5. High-strength steel at 20 °C (uniaxial test)	428.5	554.9	+ 30%		
DS-6. High-strength steel at 600 °C (uniaxial test)	177.4	247.7	+ 40%		

It is interesting to note that, for Data Set No. 2, which is shown in bold in Table 6 (Appendix A), another comparison can be made between our result and a 3p Weibull fit to the same data by Duffy, *et al*. [24], who used a nonlinear regression technique proposed by Margetson and Cooper [27]. The three parameters estimated by Duffy, *et al*. [24] differ considerably from ours (given in Appendix C) as shown in Table 5:

**Table 5:** Comparison of the estimated values of the three parameters of a Weibull distribution chosen to fit a 27 point sample data set (our Data Set No. 2) of a four-point bend fracture strength test of silicon nitride at 20 °C using the 1992 approach by Duffy, *et al* [24] and our approach described in this paper.

Modeling Approach Using a Three-Parameter Weibull Distribution Model	Parameter 1 (Location)	Parameter 2 (Scale)	Parameter 3 (Shape)
The 1992 approach by Duffy, *et al*. [24] using a nonlinear regression technique proposed by Margetson and Cooper [27].	558.1	861.6	1.68
Our three-step modeling approach using the maximum likelihood method of parameter estimation as shown in Appendix C	603.2	145.5	1.72

Since ours is based on the maximum likelihood method and four goodness-of-fit statistics criteria that include the Kolmogorov-Smirnov statistic and the Anderson-Darling statistic, both of which happened to be cited by Duffy, *et al*. [24] as keys to the next best approach after theirs using the nonlinear regression technique, we believe our work provides a direct response to their challenge, and is an improvement over their 1992 results [24].

## 7 Concluding Remarks and Future Work

A three-step approach to improve the ASTM recommended practice C1239-07 for reporting fracture strength data based on the two-parameter Weibull distribution has been formulated and applied not only to ceramics but also to a broader class of materials. Using a six-scenario goodness-of-fit test statistics ranking methodology and the classical theory of tolerance limits to analyze six sets of laboratory data, we succeeded in demonstrating that the two-parameter Weibull distribution is a poor choice to represent strength data in all six cases. In four of the six cases, the best choice among a small set of five candidate models is the three-parameter Weibull distribution, and in two, the best choice is the normal distribution. This leads us to conclude that the two-parameter Weibull, as recommended in ASTM C1239-07, is not a sound choice to represent strength data and to derive minimum strength design allowable properties, and that a statistically sounder approach such as ours is feasible and applicable to at least a large class of brittle materials as represented by the three examples of ceramics and their laboratory test data chosen in this paper. 

It is important to note that our approach and the accompanying mathematical rigor were developed specifically for very brittle materials tested in laboratory conditions. Even though we did include one example of a steel at room temperature and another at 600 °C, and our approach appeared to yield the same result as the ceramics, we believe it is premature to conclude its general applicability, since other details may be necessary for high rate loading and elevated temperature environments. By adding more material examples in our future work, we plan to answer the question whether our approach is applicable to a broader class of engineering materials in addition to brittle materials.

## 8 Appendix A: Fracture Failure Test Data of Four Engineering Materials

**Table 6:** Six sets of failure strength data for four engineering materials.

Data Set No. [Ref.] Material Name Temperature (Type of Test) Sample Size	DS-1 [6] BK-7 Glass 20 °C (Biaxial Test) 31	**DS-2**^a^ [24] Silicon Nitride, 20 °C (Four-Point Bend) 27	DS-3 [24] Silicon Nitride, 20 °C (Biaxial Test) 32	DS-4 [25] Aluminum Oxide, 20 °C (Uniaxial Test) 30	DS-5 [26] High-Strength Steel, 20 °C (Uniaxial Test) 21	DS-6 [26] High-Strength Steel, 600 C (uniaxial test) 21
Test data no.						
1	129.83	**613.9**	549.7	307	571	258
2	143.42	**623.4**	575.5	371	578	269
3	149.33	**639.3**	587.4	380	592	270
4	158.79	**642.1**	622	393	601	276
5	160.17	**653.8**	636.7	393	603	276
6	165.83	**662.4**	639.3	402	604	279
7	167.69	**669.5**	642.6	407	612	282
8	175.82	**672.8**	646.3	409	618	288
9	175.96	**681.3**	659.3	411	625	290
10	177.89	**682**	659.6	428	629	299
11	184.03	**699**	660.4	430	630	300
1	184.58	**714.5**	661.4	434	630	310
13	184.65	**717.4**	667.8	435	636	311
14	186.51	**725.5**	668.9	437	648	312
15	190.79	**741.6**	670.8	441	660	313
16	206.16	**744.9**	684.8	445	672	313
17	214.5	**751**	686.2	445	676	316
18	228.91	**761.7**	691.3	449	692	317
19	232.57	**763.9**	693.8	455	693	330
20	232.78	**774.2**	698.1	462	698	338
21	233.67	**791.6**	706.9	465	736	366
22	239.67	**795.2**	718.1	466		
23	246.5	**829.8**	718.8	480		
24	247.6	**838.4**	726.4	485		
25	254.98	**856.4**	732.2	486		
26	255.67	**868.3**	738.1	499		
27	255.74	**882.9**	748.2	499		
28	272.9		771.5	500		
29	303.69		780.7	543		
30	312.28		786.3	562	30	
31	312.9		796.2			
32			811.6			
(Note: Unit is MPa.)						

^a^ Data Set No. 2 (DS-2) is displayed in bold to bring attention to its association with a statement we made in Sec. 6 regarding a comparison of two approaches to a statistical fit of the data set.

## 9 Appendix B: Raw Goodness-of-Fit Statistics before Normalization for Six Data Sets

**Table 7:** Data Sets DS-1, DS-2, and DS-3: Goodness-of-fit statistics for five candidate models.

Goodness-of-Fit (GoF) Criterion and Parameter Estimation (PE) Method Combination (Combo) Scenario	GoF/PE Combo No.	Model 1 Normal	Model 2 Two- Parameter Weibull	Model 3 Three- Parameter Weibull	Model 4 Two- Parameter Lognormal	Model 5 Three- Parameter Lognormal
DS-1. BK-7 glass at 20 °C (biaxial test)						
CS-ML. Chi square (CS)-ML method 1 combo	CS-1	13.72	13.70	10.61	11.65	11.91
AD-ML. Anderson-Darling (AD)-ML method 1 combo	AD-1	0.532	0.597	0.338	0.389	0.398
KS-ML. Kolmorogov-Smirnov (KS)- ML method-1 combo	KS-1	0.151	0.153	0.117	0.122	0.129
AD-PC. Anderson-Darling (AD)- PPCC method 2 combo	AD-2	0.513	N.A.^a^	0.318	N.A.	0.374
KS-PC. Kolmorogov-Smirnov (KS)- PPCC method 2 combo	KS-2	0.149	N.A.	0.114	N.A.	0.124
PC-PC. PPCC (PC) criterion- PPCC method 2 combo	PC-2	0.980	N.A.	0.988	N.A.	0.986
						
DS-2. Silicon nitride at 20 °C (four-point bend test)						
CS-ML. Chi square (CS)-ML method 1 combo	CS-1	5.372	7.065	3.692	4.761	4.522
AD-ML. Anderson-Darling (AD)-ML method 1 combo	AD-1	0.313	0.539	0.197	0.244	0.226
KS-ML. Kolmorogov-Smirnov (KS)- ML method-1 combo	KS-1	0.115	0.112	0.082	0.109	0.092
AD-PC. Anderson-Darling (AD)- PPCC method 2 combo	AD-2	0.289	N.A.	0.153	N.A.	0.184
KS-PC. Kolmorogov-Smirnov (KS)- PPCC method 2 combo	KS-2	0.110	N.A.	0.086	N.A.	0.095
PC-PC. PPCC (PC) criterion- PPCC method 2 combo	PC-2	0.986	N.A.	0.994	N.A.	0.991
						
DS-3. Silicon nitride at 20 °C (biaxial test)						
CS-ML. Chi square (CS)-ML method 1 combo	CS-1	4.477	6.719	5.476	4.078	4.594
AD-ML. Anderson-Darling (AD)-ML method 1 combo	AD-1	0.232	0.500	1.000	0.232	0.231
KS-ML. Kolmorogov-Smirnov (KS)- ML method-1 combo	KS-1	0.080	0.110	1.000	0.086	0.079
AD-PC. Anderson-Darling (AD)- PPCC method 2 combo	AD-2	0.237	N.A.	0.263	N.A.	0.237
KS-PC. Kolmorogov-Smirnov (KS)- PPCC method 2 combo	KS-2	0.087	N.A.	0.092	N.A.	0.089
PC-PC. PPCC (PC) criterion- PPCC method 2 combo	PC-2	0.992	N.A.	0.991	N.A.	0.990

^a^ N.A. is not applicable.

## **9. Appendix B** (Continued)

**Table 8:** Data Sets DS-4, DS-5, and DS-6: Goodness-of-fit statistics for five candidate models.

Goodness-of-Fit (GoF) Criterion and Parameter Estimation (PE) Method Combination (Combo) Scenario	GoF/PE Combo No.	Model 1 Normal	Model 2 Two- Parameter Weibull	Model 3 Three- Parameter Weibull	Model 4 Two- Parameter Lognormal	Model 5 Three- Parameter Lognormal
DS-4. Aluminum oxide at 20 °C (uniaxial test)						
CS-ML. Chi square (CS)-ML method 1 combo	CS-1	0.562	1.591	0.725	0.536	10.00
AD-ML. Anderson-Darling (AD)-ML method 1 combo	AD-1	0.232	0.472	1.000	0.293	1.000
KS-ML. Kolmorogov-Smirnov (KS)- ML method-1 combo	KS-1	0.080	0.106	1.000	0.102	1.000
AD-PC. Anderson-Darling (AD)- PPCC method 2 combo	AD-2	0.255	N.A.	0.309	N.A.	0.271
KS-PC. Kolmorogov-Smirnov (KS)- PPCC method 2 combo	KS-2	0.082	N.A.	0.083	N.A.	0.097
PC-PC. PPCC (PC) criterion- PPCC method 2 combo	PC-2	0.985	N.A.	0.983	N.A.	0.983
						
DS-5. High-strength steel at 20 °C (uniaxial test)						
CS-ML. Chi square (CS)-ML method 1 combo	CS-1	2.516	3.652	2.099	2.354	2.340
AD-ML. Anderson-Darling (AD)-ML method 1 combo	AD-1	0.328	0.603	0.187	0.273	0.188
KS-ML. Kolmorogov-Smirnov (KS)- ML method-1 combo	KS-1	0.147	0.173	0.090	0.136	0.095
AD-PC. Anderson-Darling (AD)- PPCC method 2 combo	AD-2	0.317	N.A.^a^	0.166	N.A.	0.190
KS-PC. Kolmorogov-Smirnov (KS)- PPCC method 2 combo	KS-2	0.145	N.A.	0.100	N.A.	0.112
PC-PC. PPCC (PC) criterion- PPCC method 2 combo	PC-2	0.983	N.A.	0.995	N.A.	0.992
						
DS-6. High-strength steel at 600 °C (uniaxial test)						
CS-ML. Chi square (CS)-ML method 1 combo	CS-1	1.320	2.449	10.00	1.075	10.00
AD-ML. Anderson-Darling (AD)-ML method 1 combo	AD-1	0.343	0.616	0.304	0.309	0.327
KS-ML. Kolmorogov-Smirnov (KS)- ML method-1 combo	KS-1	0.123	0.168	0.151	0.131	0.159
AD-PC. Anderson-Darling (AD)- PPCC method 2 combo	AD-2	0.343	N.A.	0.283	N.A.	0.293
KS-PC. Kolmorogov-Smirnov (KS)- PPCC method 2 combo	KS-2	0.129	N.A.	0.137	N.A.	0.139
PC-PC. PPCC (PC) criterion- PPCC method 2 combo	PC-2	0.977	N.A.	0.984	N.A.	0.985

^a^ N.A. is not applicable.

## 10 Appendix C: Minimum Strengths of Silicon Nitride (20 °C) of Data Set DS-2

**Table 9:** Estimates of minimum strength at laboratory- and full-scale sizes for five candidate models based on Data Set DS-2 (silicon nitride) at 20 °C (four-point bend test).

	Model 1 Normal	Model 2 2p Weibull	Model 3 3p Weibull	Model 4 2p lognormal	Model 5 3p lognormal
Laboratory-Scale					
Composite normalized GoF statistic (metric 1) (Ranges from 0 to 1, worst to best.)	0.19	0.03 (worst)	1.0 (best)	0.58	0.73
Parameter 1 (Location) Point estimate of location, Standard deviation of location	733.2 15.0	None. None.	603.2 18.5	None. None.	470.9 125.2
One-sided 95% confidence minimum strength at sample scale = (lower limit, point estimate, upper limit)	(546.6, 605.4, 643.0)	(512.6, 573.0, 633.4)	(572.8, 603.2, 633.6)	(566.7, 613.6, 645.6)	(264.9, 470.9, 676.9)
Parameter 2 (Scale) Point estimate of scale, Standard deviation of scale	77.7 10.8	768.5 15.4	145.5 29.6	729.3 14.9	251.3 131.6
Parameter 3 (Shape) Point estimate of shape, Standard deviation of shape	None. None.	10.1 1.52	1.72 0.415	0.105 0.03	0.296 0.159
Full-Scale Size					
95% confidence, 99% coverage A-basis design allowable (AbDA) Uncertainty (metric 2) (Note: Small is good.)	11%	15% (worst)	4% (best)	13%	7%
95% confidence, 99% coverage minimum strength at full scale (lower tolerance limit or A-basis of design, mean estimate, upper tolerance limit)	(476.8, 552.4, 598.6)	(414.2, 487.7, 561.2)	(590.5, 613.2, 635.9)	(515.7, 571.2, 667.3)	(558.2, 597.1, 636.1)
95% confidence, 90% coverage minimum strength at full scale (lower tolerance limit or B-basis of design, mean estimate, upper tolerance limit)	(583.0, 633.6, 667.4)	(562.2, 615.2, 668.2)	(621.9, 642.4, 662.9)	(595.3, 637.5, 667.3)	(616.7, 642.9, 669.0)

## 11 Appendix D: Minimum Strengths of Silicon Nitride (20 °C) of Data Set DS-3

**Table 10:** Estimates of minimum strength at laboratory- and full-scale sizes for five candidate models based on Data Set DS-3 (silicon nitride) at 20 °C (biaxial test).

	Model 1 Normal	Model 2 2p Weibull	Model 3 3p Weibull	Model 4 2p lognormal	Model 5 3p lognormal
Laboratory-Scale.					
Composite normalized GoF statistic (metric 1) (Ranges from 0 to 1, worst to best.)	0.97 (best)	0.54	0.16 (worst)	(Disqualified. See Table 3 for more details.)	0.73
Parameter 1 (Location) Point estimate of location, Standard deviation of location	688.7 11.2	None. None.	402.5 146.9	None. None.	-3096. 33279.
One-sided 95% confidence minimum strength at sample scale = (lower limit, point estimate, upper limit)	(542.2, 584.9, 613.3)	(514.4, 560.1, 605.8)	(160.9, 402.5, 644.1)	(553.3, 589.0 614.1)	Negative Value
Parameter 2 (Scale) Point estimate of scale, Standard deviation of scale	63.1 8.0	717.1 11.1	310.9 150.6	685.8 11.3	3784. (Not available.)
Parameter 3 (Shape) Point estimate of shape, Standard deviation of shape	None. None.	12.02 1.66	5.21 2.65	0.092 0.012	0.016 (Not available.)
Full-Scale Size.					
95% confidence, 99% coverage A-basis design allowable (AbDA) Uncertainty (metric 2) (Note: Small is good.)	8% (best)	12% (worst)	12% (worst)	(Disqualified. See Table 3 for more details.)	10%
95% confidence, 99% coverage minimum strength at full scale (lower tolerance limit or A-basis of design, mean estimate, upper tolerance limit)	(487.1, 541.9, 576.8)	(432.1, 489.1, 546.1)	(469.3, 531.2, 593.0)	(510.3, 553.0, 582.1)	(491.6, 546.4, 601.2)
95% confidence, 90% coverage minimum strength at full scale (lower tolerance limit or B-basis of design, mean estimate, upper tolerance limit)	(571.0, 607.8, 633.3)	(555.0, 594.7, 634.3)	(572.2, 604.4, 636.6)	(577.1, 609.1, 632.3)	(580.4, 609.4, 638.3)

## 12 Appendix E: Minimum Strengths of Aluminum Oxide (20 °C) of Data Set DS-4

**Table 11:** Estimates of minimum strength at laboratory- and full-scale sizes for five candidate models based on Data Set DS-4 (aluminum oxide) at 20 °C (uniaxial test).

	Model 1 Normal	Model 2 2p Weibull	Model 3 3p Weibull	Model 4 2p lognormal	Model 5 3p lognormal
Laboratory-Scale					
Composite normalized GoF statistic (metric 1) (Ranges from 0 to 1, worst to best.)	1.0 (best)	0.88	0.32	0.97	0.12 (worst)
Parameter 1 (Location) Point estimate of location, Standard deviation of location	444.0 9.5	None. None.	258.3 67.1	None. None.	-1331. (Not available.)
One-sided 95% confidence minimum strength at sample scale = (lower limit, point estimate, upper limit)	(321.6, 358.3, 382.4)	(301.3, 338.3, 375.4)	(147.9, 258.3, 368.6)	(332.2, 361.7, 382.4)	(Not Available.)
Parameter 2 (Scale) Point estimate of scale, Standard deviation of scale	52.1 6.8	466.6 9.7	204.9 70.4	440.9 9.8	1775. (Not available.)
Parameter 3 (Shape) Point estimate of shape, Standard deviation of shape	None. None.	9.24 1.32	4.00 1.53	0.12 0.016	0.29 (Not available.)
Full-Scale Size.					
95% confidence, 99% coverage A-basis design allowable (AbDA) Uncertainty (metric 2) (Note: Small is good.)	12%	16%	13%	9%	57%
95% confidence, 99% coverage minimum strength at full scale (lower tolerance limit or A-basis of design, mean estimate, upper tolerance limit)	(275.7, 322.9, 352.5)	(239.2, 283.6, 328.0)	(282.1, 323.2, 364.3)	(298.7, 333.1, 356.8)	(Not Available.)
95% confidence, 90% coverage minimum strength at full scale (lower tolerance limit or B-basis of design, mean estimate, upper tolerance limit)	(345.6, 377.3, 398.9)	(333.0, 365.8, 398.5)	(350.2, 375.0, 399.8)	(351.2, 377.8, 397.2)	(Not available.)

## 13 Appendix F: Minimum Strengths of a High-Strength Steel (20 °C) of Data Set DS-5

**Table 12:** Estimates of minimum strength at laboratory- and full-scale sizes for five candidate models based on Data Set DS-5 (high-strength steel) at 20 °C (uniaxial test).

	Model 1 Normal	Model 2 2p Weibull	Model 3 3p Weibull	Model 4 2p lognormal	Model 5 3p lognormal
Laboratory-Scale					
Composite normalized GoF statistic (metric 1) (Ranges from 0 to 1, worst to best.)	0.28	0 (worst)	1.0 (best)	0.69	0.85
Parameter 1 (Location) Point estimate of location, Standard deviation of location	538.3 9.5	None. None.	563.5 12.4	None. None.	494.2 77.8
One-sided 95% confidence minimum strength at sample scale = (lower limit, point estimate, upper limit)	(527.8, 566.8, 590.2)	(496.6, 540.2, 583.9)	(543.2, 563.5, 583.9)	(536.8, 570.2, 591.2)	(366.2, 494.2, 622.3)
Parameter 2 (Scale) Point estimate of scale, Standard deviation of scale	43.4 6.9	658.8 10.1	83.9 19.0	636.9 9.4	137.9 81.8
Parameter 3 (Shape) Point estimate of shape, Standard deviation of shape	None. None.	15.0 2.55	1.80 0.53	0.067 0.011	0.296 0.181
Full-Scale Size					
95% confidence, 99% coverage A-basis design allowable (AbDA) Uncertainty (metric 2) (Note: Small is good.)	7%	12% (worst)	3% (best)	6%	4%
95% confidence, 99% coverage minimum strength at full scale (lower tolerance limit or A-basis of design, mean estimate, upper tolerance limit)	(486.8, 537.2, 565.9)	(428.5, 484.5, 540.5)	(554.9, 570.1, 585.2)	(503.8, 544.7, 569.4)	(539.2, 563.5, 587.7)
95% confidence, 90% coverage minimum strength at full scale (lower tolerance limit or B-basis of design, mean estimate, upper tolerance limit)	(549.1, 582.6, 603.7)	(529.4, 566.9, 604.3)	(574.2, 587.6, 601.0)	(554.8, 584.3, 605.7)	(572.3, 588.6, 604.9)

## 14 Appendix G: Minimum Strengths of a High-Strength Steel (600 °C) of Data Set DS-6

**Table 13:** Estimates of minimum strength at laboratory- and full-scale sizes for five candidate models based on Data Set DS-6 (high-strength steel) at 600 °C (uniaxial test).

	Model 1 Normal	Model 2 2p Weibull	Model 3 3p Weibull	Model 4 2p lognormal	Model 5 3p lognormal
Laboratory-Scale					
Composite normalized GoF statistic (metric 1) (Ranges from 0 to 1, worst to best.)	0.64	0.28	0.58	(Disqualified. See Table 3 for more details.)	0.49
Parameter 1 (Location) Point estimate of location, Standard deviation of location	300.6 5.7	None. None.	252.6 8.4	None. None.	197.5 68.6
One-sided 95% confidence minimum strength at sample scale = (lower limit, point estimate, upper limit)	(234.0, 257.5, 271.6)	(215.6, 241.1, 266.7)	(238.8, 252.6, 266.5)	(240.7, 260.1, 272.4)	(84.4, 197.5, 310.6)
Parameter 2 (Scale) Point estimate of scale, Standard deviation of scale	26.2 4.1	312.7 6.3	54.0 12.0	299.6 5.7	100.0 71.1
Parameter 3 (Shape) Point estimate of shape, Standard deviation of shape	None. None.	11.4 1.9	1.91 0.56	0.086 0.014	0.248 0.18
Full-Scale Size					
95% confidence, 99% coverage A-basis design allowable (AbDA) Uncertainty (metric 2) (Note: Small is good.)	10%	15%	4% (best)	8%	6%
95% confidence, 99% coverage minimum strength at full scale (lower tolerance limit or A-basis of design, mean estimate, upper tolerance limit)	(209.3, 239.7, 256.9)	(177.4, 209.1, 240.7)	(247.7, 257.7, 267.7)	(222.0, 245.3, 259.6)	(237.3, 253.7, 270.1)
95% confidence, 90% coverage minimum strength at full scale (lower tolerance limit or B-basis of design, mean estimate, upper tolerance limit)	(246.8, 267.0, 279.8)	(234.6, 256.8, 279.0)	(260.9, 269.6, 278.4)	(251.1, 268.3, 279.8)	(259.8, 270.3, 280.8)
